# Increased risk of non-hematological cancer in young patients with aortic stenosis: a retrospective cohort study

**DOI:** 10.1186/s40959-021-00123-w

**Published:** 2021-10-25

**Authors:** Walid Saliba, Tamir Bental, Yaron Shapira, Shmuel Schwartzenberg, Alex Sagie, Moti Vaturi, Salim Adawi, Alexander Fuks, Ami Aronheim, Avinoam Shiran

**Affiliations:** 1 Department of Community Medicine and Epidemiology, Lady Davis Carmel Medical Center, Haifa, Israel; 2 The Ruth and Bruce Rappaport Faculty of Medicine, Technion, Israel Institute of Technology, Haifa, Israel; 3 Department of Cardiology, Rabin Medical Center, Petah Tikva, Israel; 4 Department of Cardiology, Lady Davis Carmel Medical Center, 7 Michal Street, 3436212 Haifa, Israel; 5 Cell Biology and Cancer Science, Technion, Israel Institute of Technology, Haifa, Israel

**Keywords:** Aortic stenosis, Cancer, Age, Risk

## Abstract

**Background:**

We have previously reported an increased risk for non-hematological malignancies in young patients with moderate or severe aortic stenosis (AS). These findings were the result of a post-hoc analysis from a large echocardiography database and needed verification. Our aim was to determine, using a different study population, whether young patients with AS are at increased risk for cancer.

**Methods:**

A large echocardiographic database was used to identify patients (age ≥ 20 years) with moderate or severe AS (study group) and patients without aortic stenosis (comparative group). The new occurrence of non-hematological malignancies was determined after the index date (first echo with moderate or severe AS or first recorded echo in the control group).

**Results:**

The final study group included 7013 patients with AS and 98,884 without AS. During a median follow-up of 6.9 years (3.0–11.1) there were 10,705 new cases of non-hematological cancer. The crude incidence rate of cancer was higher in AS compared to non-AS patients (22.3 vs. 13.7 per 1000 patient-year, crude HR 1.58 (95%CI 1.46–1.71). After adjustment for relevant covariates, there was no difference between groups (HR 0.93, 95% CI 0.86–1.01). Only patients in the lowest age quartile (20–49.7 years), had an increased adjusted risk of cancer (HR 1.91, 95%CI 1.08–3.39). The HR for the risk of cancer associated with AS was inversely proportional to age (*P* < 0.001 for the interaction between AS and age).

**Conclusions:**

Young patients with moderate or severe AS may have an increased risk for cancer. Cancer surveillance should be considered for young patients with AS.

## Introduction

Reverse cardio-oncology has been recently proposed as a new field, dealing with inherent cancer risk in patients with cardiovascular disease [1]. Cancer and cardiovascular disease share similar risk factors, including smoking, obesity, diabetes mellitus, hyperlipidemia and a sedentary lifestyle, which may partly explain this phenomenon [2, 3]. Increased risk of cancer has been reported in patients with heart failure (HF) [4–6]. Compared to patients without HF, community patients with HF were found to have a 68% higher adjusted risk of developing cancer which was associated with higher mortality, during an average follow-up of 7.7 years [4]. Similarly, patients who developed HF after acute myocardial infarction had an increased adjusted risk of cancer [5]. In a Danish population study, increased risk of cancer was reported in all age groups of outpatients with chronic HF, but a surveillance bias could not be ruled out [6]. To determine whether causal relationship exists between HF and the development of cancer, Meijers et al. used a mouse model of myocardial infarction and HF, and showed increased intestinal tumor growth, proportional to the amount of scar tissue in the left ventricle and inversely proportional to left ventricular ejection fraction [7]. This effect was mediated by cardiac secreted proteins. In another study, myocardial infarction was shown to accelerate breast cancer progression and mortality in mice and humans through an epigenetic switch in monocytes to an immunosuppressive phenotype [8].

We have previously shown in a transverse aortic constriction (TAC) murine model, that early cardiac remodeling secondary to pressure overload, without HF, is associated with increased risk of cancer [9]. In this model, cardiac remodeling was associated with increased tumor growth and metastasis seeding, which was suggested to be mediated by periostin, a matrix protein that enhances cancer cell proliferation. In that study, using a large retrospective echocardiographic database and the national cancer registry we have shown that moderate or severe aortic stenosis (AS) is associated with a significantly increased risk of non-hematologic malignancies in patients younger than 60 years old, but not in older patients [9]. Our findings suggested that age may modify the effect of AS on non-hematologic malignancies. These findings were the result of post-hoc analysis and needed confirmation by an independent data set. Our aim in the present study, was to examine the effect of AS on the risk of non-hematologic malignancies in different age groups, using data from a different patient population.

## Methods

### Source of data

In the present study we used the echocardiography database of a large tertiary hospital, the Rabin Medical Center from the Clalit Health Services (CHS), which includes both inpatients and outpatients. CHS hospitals provide health services for all patients, including patients from other health maintenance organizations (HMO). This analysis was restricted to CHS members, for whom we have full access to clinical data including outcomes data and other relevant confounders. CHS provides inclusive health care for more than half of the Israeli population. Health care coverage in Israel is mandatory according to the National Health Insurance Law (1995) and is provided by four groups akin to non-profit health HMO. All members of the different HMOs have a similar health insurance plan and similar access to health services. The electronic medical record database of CHS includes data from multiple sources: records of primary care physicians, community specialty clinics, hospitalizations, laboratories, and pharmacies. A registry of chronic disease diagnoses is compiled from these data sources. Diagnoses are captured in the registry by diagnosis-specific algorithms, employing International Classification of Diseases Ninth revision (ICD-9) code reading, laboratory test results and disease-specific drug usage. A record is kept of the data-sources and dates used to establish the diagnosis, with the earliest recorded date, from any source, considered to be the defining date of diagnosis. This process of capturing data into the registry reassures that a specific type of cancer diagnosed after the index date is not a recurrence or continuation of an original cancer diagnosed before the index date. A number of high-quality, population-based studies have been conducted based on the data retrieved from CHS database [10–12].

The electronic medical records database of the CHS is periodically updated with data from the Israel National Cancer Registry (INCR). Cancer reporting by hospitals, pathology and cytology laboratories and other health care providers has been mandatory since 1982. The INCR collects data on all malignant, in-situ and invasive tumors (excluding basal and squamous cell carcinomas of the skin). The completeness of the INCR is estimated at 96.8% for invasive solid malignancies [13]. The current retrospective cohort study comprised only invasive non-hematological malignancies (invasive solid tumors).

### Selection of study population

Eligible patients were CHS adult members age ≥ 20 years who underwent echocardiography at Rabin Medical Center, between January 1st, 2005 and December 31st, 2019, and were found to have either no documentation of any degree of AS or at least one test showing moderate or severe AS. Patients were excluded if they were not members of CHS, and if they had coronary artery bypass surgery, or they had aortic or mitral valve replacement before the index date. The first dated echo showing at least moderate AS was used to define the index date of the exposure group (AS group), and the first dated echo of patients without evidence of any degree of AS was used to define the index date of the comparative group (no AS). The two groups were followed from the index date through October 30, 2020, for the occurrence of non-hematological cancer.

The study was approved by the Institutional Review Board (IRB) and conducted in accordance with the Declaration of Helsinki.

### Echocardiography and study variables

A complete transthoracic echocardiographic study was performed using standard views and techniques according to established guidelines [14]. The diagnosis of AS was based on 2-dimmensional echocardiographic aortic valve anatomy, trans-aortic Doppler gradients and aortic valve area (AVA), with moderate or severe AS defined as AVA ≤1.5 cm^2^, and no AS defined as AVA > 2 cm^2^ or maximal aortic velocity < 2.5 m/sec [15]. AVA was calculated using the continuity equation.

In addition, for each patient the following baseline data were retrieved from the computerized database of the CHS: demographic and other descriptive variables, smoking status (ever, never), alcohol abuse, obesity, socioeconomic status (SES, based on the SES score of the clinic neighborhood as defined by the Israeli Central Bureau of Statistics), presence of selected chronic medical conditions, cancer risk factors and medication use of selected drug categories. SES had missing values; hence, this variable was used in the analyses as categorical variables that include a category of missing values.

The study outcome was defined as the occurrence of invasive non-hematologic malignancies at all sites, based on combined data from multiple sources in the CHS databases and data from the Israeli National Cancer Registry (INCR).

### Statistical methods

Statistical analyses were performed using IBM SPSS Statistics 24.0 (IBM, New York, NY), and SAS version 9.3 software. For all analyses, *P* < 0.05 for the 2-tailed tests was considered statistically significant. Continuous variables are summarized with mean ± SD, and categorical variables are presented as numbers and proportions. Comparisons of baseline characteristics, between patients with AS and patients without AS, were performed using the chi-square test for categorical variables and using student t-test for continuous variable.

The annualized incidence rate of invasive solid tumor was estimated by dividing the number of incident cases by the total follow up time and was expressed as number per 1000 person-year of observation. Because time to invasive non-hematologic malignancies faces the competing risk of mortality, we used cumulative incidence function (CIF) to estimate the distribution of time to reach invasive non-hematologic malignancies [16]. The Gray’s test for equality of CIF was used to compare curves of patients with AS and without AS. Cox proportional hazard regression models were used to estimate the crude and the adjusted hazard ratio (HR) for the association between AS and non-hematologic malignancies. The main Cox multivariable model was adjusted for age (continuous variable), sex, ethnicity, socioeconomic status, smoking, alcohol abuse, obesity, diabetes mellitus, previous history of cancer and aspirin and statin use. An interaction between AS status and age was tested by including an interaction factor of both variables into the multivariable Cox regression model. In addition, we performed the following analyses: i) To assess the robustness of our results we repeated the multivariable analysis and performed further adjustment for additional variables that were not accounted for in the main analysis, including; heart failure, ischemic heart disease, peripheral vascular disease, previous stroke, atrial fibrillation, and year of study entry. ii) To avoid possible biases, we restricted the analysis to patients without a history of cancer, since they can retain their propensity for other cancer, iii) A one-year landmark analysis was performed in order to assess the effect of possible surveillance bias shortly after AS diagnosis. This analysis excluded patients with cancer diagnosed during the first year, and was restricted to patients with greater than 1 year of follow-up.

## Results

### Study population

**Fig. 1:**
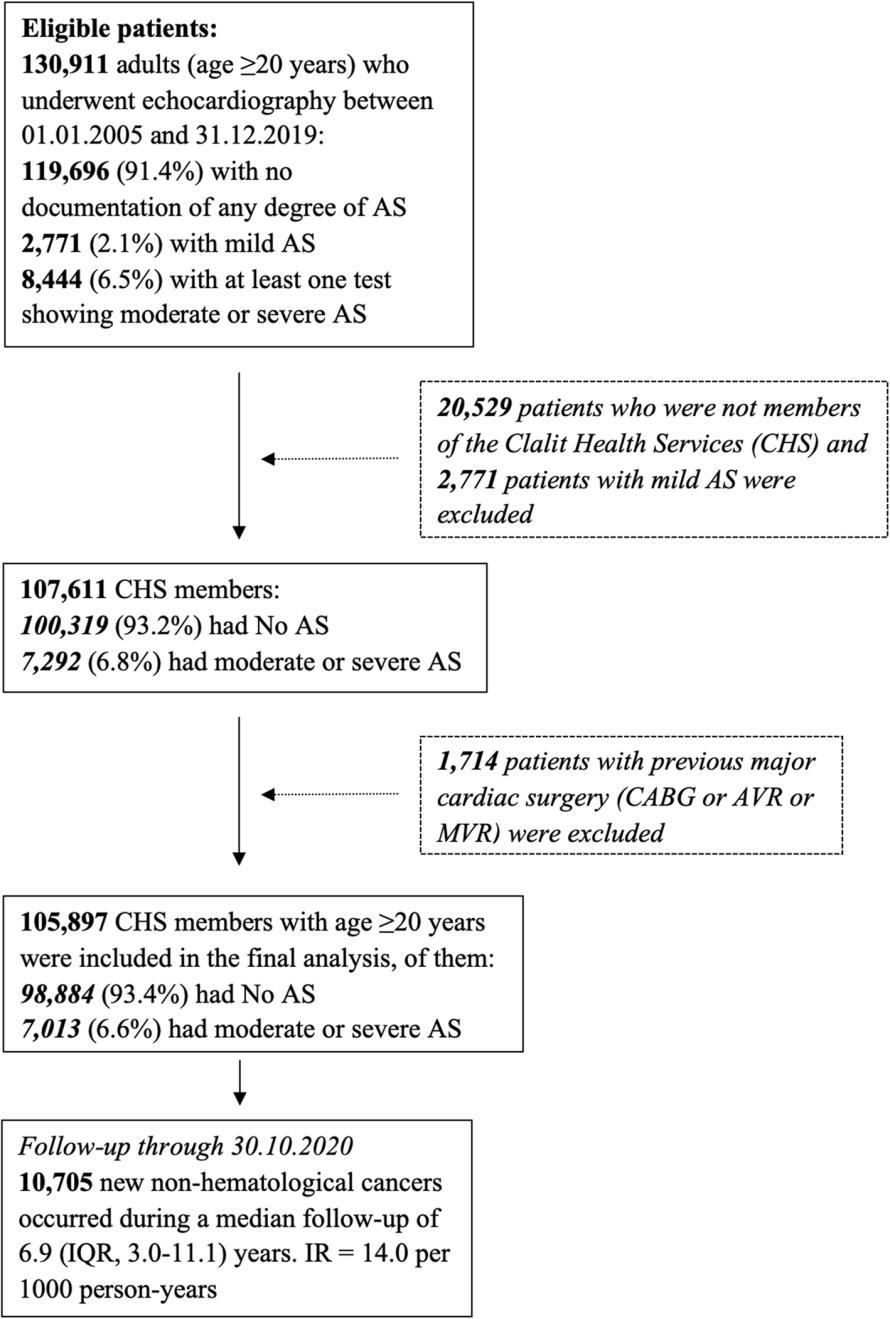
Study population selection flowchart. AS = aortic stenosis, AVR = aortic valve replacement, CABG = coronary artery bypass grafting, IQR = interquartile range, IR = incidence rate, MVR = mitral valve replacement

The final study population included 105,897 patients. AS (moderate or severe) was present in 7013 patients, and 98,884 did not have AS (Fig. 1). The study group demographic and clinical characteristics are summarized in Table 1. Patients with AS were older and more likely to have previous cancer, comorbidities, HF and cardiovascular disease. Severe AS was present in 4125/7013 patients with AS (58.8%). Moderate or severe AS was present in 183/26,476 (0.7%) patients in the lowest age quartile (20–49.7 years) and 4662/26,469 (18%) of patients in the highest age quartile (> 74.7 years).

**Table 1:** Baseline demographic and clinical characteristics of the entire study population according to AS status

	All *(n = 105,897)*	No AS *(n = 98,884)*	Moderate or severe AS *(n = 7013)*	***P*** value
**Age**	61.1 ± 17.5	60.0 ± 17.3	77.1 ± 11.4	< 0.001
**Sex**				0.883
Males	52,655 (49.7%)	49,162 (49.7%)	3493 (49.8%)	
Females	53,242 (50.3%)	49,722 (50.3%)	3520 (50.2%)	
**Ethnicity**				< 0.001
Arabs	7314 (6.9%)	7008 (7.1%)	306 (4.4%)	
Jews	98,583 (93.1%)	91,876 (92.9%)	6707 (95.6%)	
**SES**^**a**^				0.458
Low	17,464 (16.5%)	16,291 (16.5%)	1173 (16.7%)	
Middle	53,310 (50.3%)	49,820 (50.4%)	3481 (49.6%)	
High	33,175 (31.3%)	30,936 (31.3%)	2239 (32.0%)	
**Comorbidities and risk factors**				
Smoking	38,328 (36.2%)	35,833 (36.2%)	2495 (35.6%)	0.266
Alcohol abuse	1052 (1.0%)	992 (1.0%)	60 (0.9%)	0.238
Obesity	25,451 (24.0%)	23,171 (23.4%)	2280 (32.5%)	< 0.001
Previous cancer	18,725 (17.7%)	16,957 (17.1%)	1768 (25.2%)	< 0.001
Diabetes	26,830 (25.3%)	23,976 (24.2%)	2854 (40.7%)	< 0.001
Hypertension	54,362 (51.3%)	48,681 (49.2%)	5681 (81.0%)	< 0.001
Congestive heart failure	11,211 (10.6%)	9095 (9.2%)	2116 (30.2%)	< 0.001
Previous myocardial infarction	20,607 (19.5%)	18,275 (18.5%)	2332 (33.3%)	< 0.001
Ischemic heart disease	31,523 (29.8%)	27,659 (28.0%)	3864 (55.1%)	< 0.001
Peripheral vascular disease	9756 (9.2%)	8095 (8.2%)	1661 (23.7%)	< 0.001
Previous stroke	11,419 (10.8%)	10,060 (10.2%)	1359 (19.4%)	< 0.001
Atrial fibrillation	13,087 (12.4%)	10,980 (11.1%)	2107 (30.0%)	< 0.001
**Medications use**				
Aspirin	34,982 (33.0%)	31,334 (31.7%)	3648 (52.0%)	< 0.001
Statin	44,690 (42.2%)	40,014 (40.5%)	4676 (66.7%)	< 0.001
Beta blockers	31,845 (30.1%)	28,248 (28.6%)	3597 (51.3%)	< 0.001
ACE-i/ARBs	39,413 (37.2%)	35,105 (35.5%)	4308 (61.4%)	< 0.001
Diuretics	21,975 (20.8%)	18,930 (19.1%)	3045 (43.4%)	< 0.001
**Echocardiography**				
LVEDD (cm)	4.60 ± 057	4.60 ± 0.56	4.60 ± 0.67	0.975
LVESD (cm)	2.84 ± 0.64	2.84 ± 0.63	2.98 ± 0.76	< 0.001
IVS (cm)	1.01 ± 0.23	0.99 ± 0.22	1.24 ± 0.20	< 0.001
PW (cm)	0.94 ± 0.19	0.92 ± 0.18	1.14 ± 0.19	< 0.001
LV MASS (gr)	156.9 ± 60.15	152.76 ± 58.00	209.22 ± 62.00	< 0.001
**As severity**				
Severe AS			4125 (58.8%)	
Moderate AS			2888 (41.2%)	
Aortic valve area (cm^2^)				
Mean ± SD			0.94 ± 0.28	
Median (IQR)			0.90 (0.70–1.13)	
Max aortic velocity (m/s)				
Mean ± SD			3.53 ± 0.83	
Median (IQR)			3.46 (2.93–4.09)	
Mean aortic gradient (mmHg)				
Mean ± SD			32.0 ± 17.6	
Median (IQR)			29.0 (20.0–42.0)	

*ACE-i/ARBs* angiotensin converting enzyme inhibitors/angiotensin II receptor blockers, *AS* aortic stenosis, *IVS* interventricular septum, *LV* left ventricular, *LVEDD* left ventricular end diastolic diameter, *LVESD* left ventricular end systolic diameter, *PW* posterior wall, *SES* socioeconomic class

^a^SES was missing in 1957 (1.8%) patients

### Risk of cancer and age

**Fig. 2:**
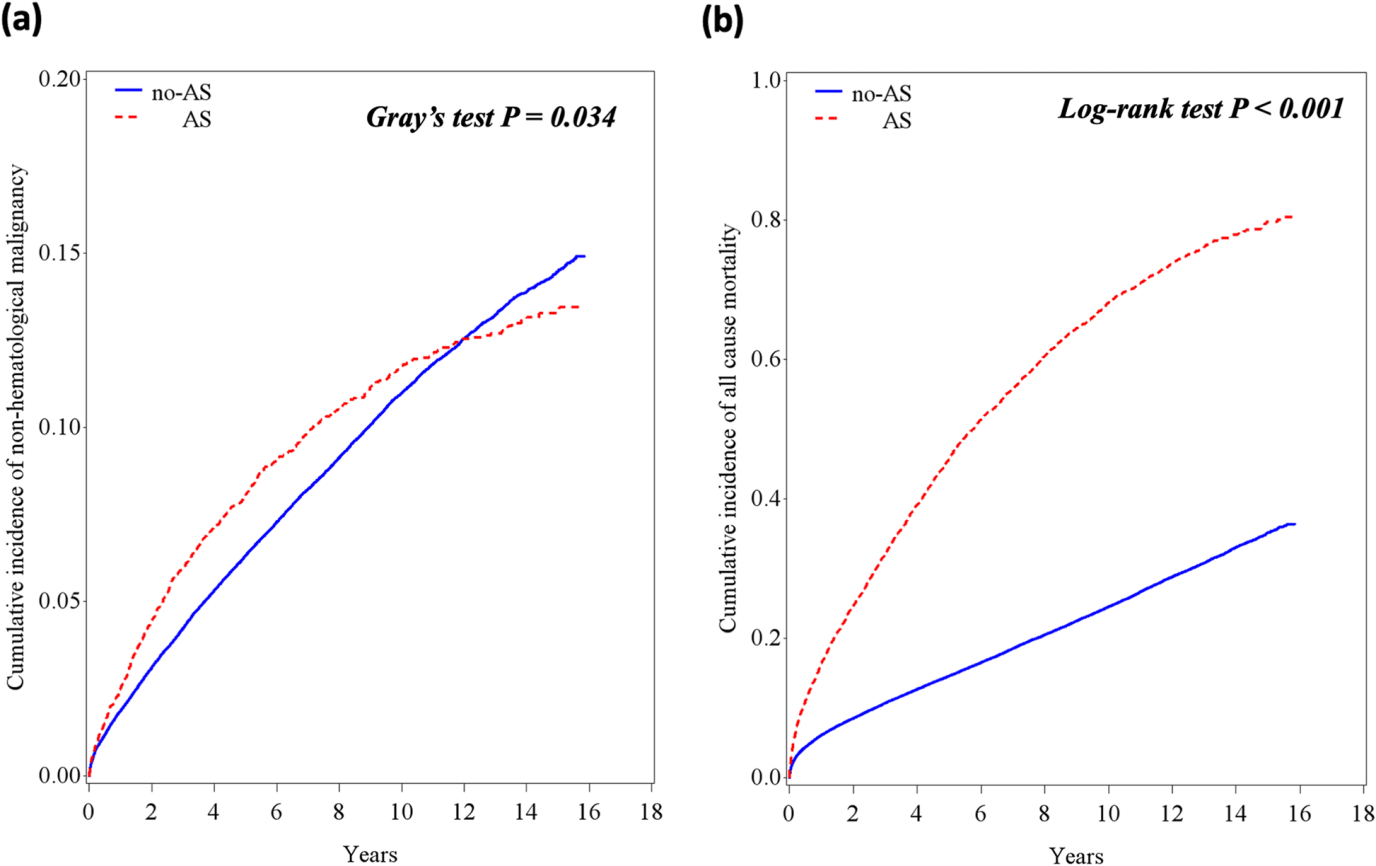
Incidence of cancer and mortality in patients with and without AS. Cumulative incidence function (CIF) for the distribution of time to non-hematological malignancies (**a**) and Kaplan-Meier curves for mortality (**b**). Death was considered a competing event for cancer (**a**). Solid line represents patients without AS and dotted line patients with AS

**Fig. 3:**
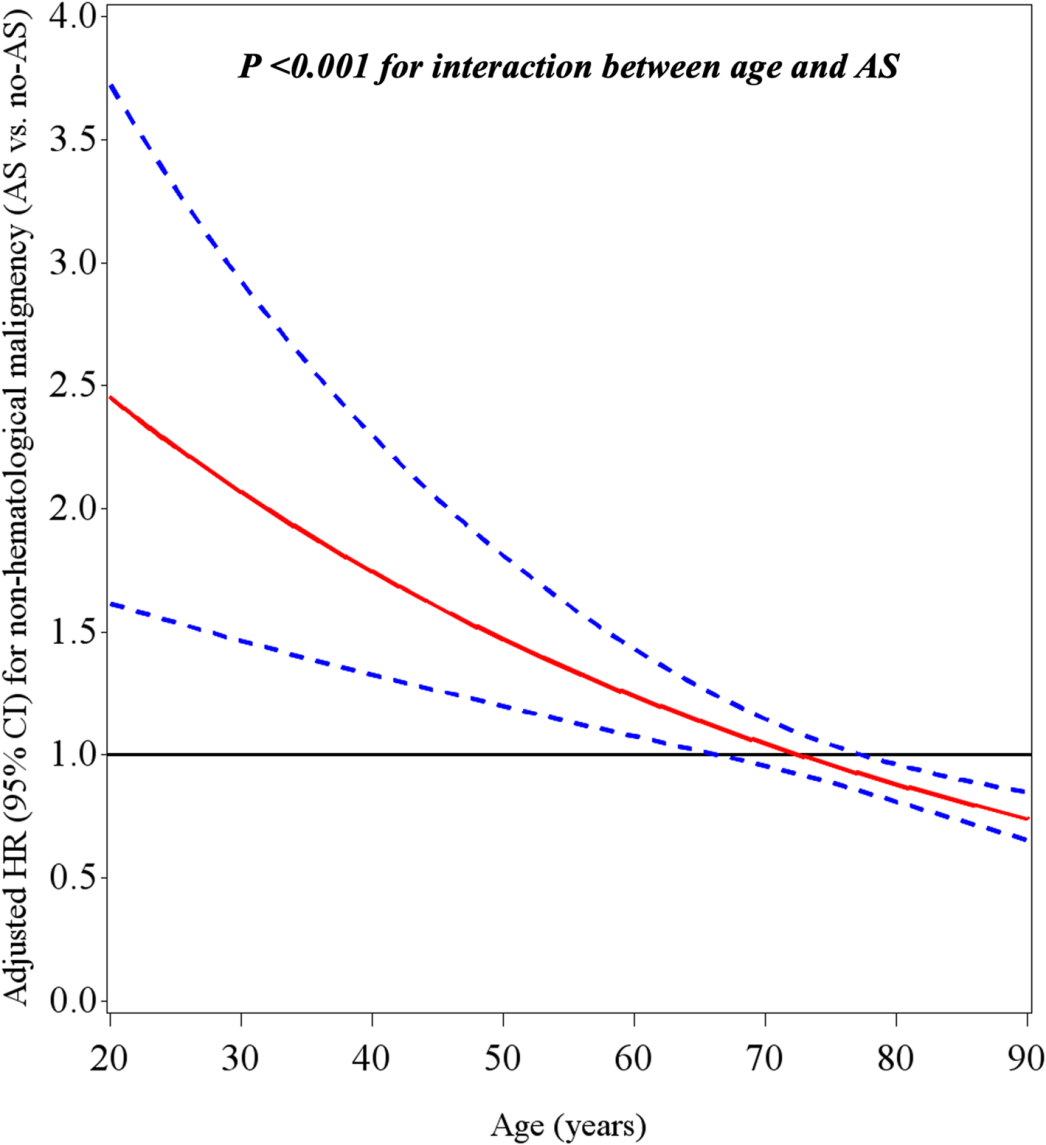
The interaction between age and AS and the risk of cancer. Adjusted HR* for non-hematological malignancies in AS as a function of age. Dotted lines represent the 95% confidence interval (CI). *Adjusted for age (continuous variable), sex, ethnicity, socioeconomic status, smoking, alcohol abuse, obesity, diabetes mellitus, previous history of cancer and aspirin and statin use. HR = hazard ratio

**Fig. 4:**
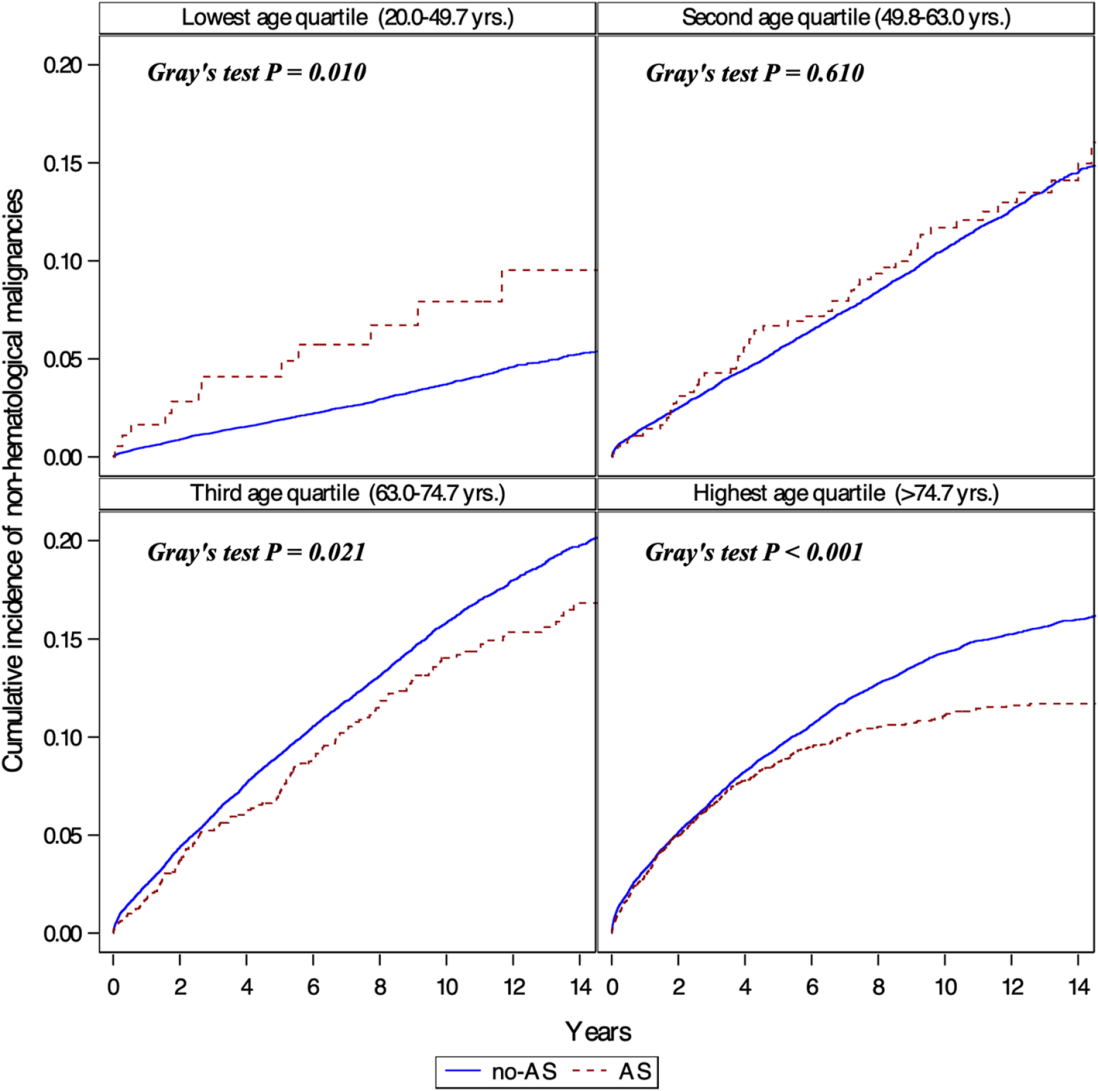
Incidence of cancer by age. Cumulative incidence function (CIF) for the distribution of time to non-hematological malignancies stratified by age quartiles. Death was considered a competing event for cancer. Increased incidence of cancer is evident only in the lowest age quartile. Solid line represents patients with no AS and dotted lines represent patients with AS

Overall, there were 10,705 new cases of non-hematological cancer during a median follow-up of 6.9 years (IQR 3.0–11.1) (Fig. 1, Table 2). The cumulative incidence of cancer and mortality among AS and non-AS patients is shown in Fig. 2. The curves diverge shortly after the index echo, showing a higher incidence of cancer and mortality in AS. However, afterward the CIF curve for non-hematological malignancy crosses between the two cohorts (Fig. 2a). Indeed, a test of the sub-distribution hazards proportionality assumption showed that this assumption was violated. The crude incidence rate of cancer was higher in AS as compared to non-AS patients (22.7 vs. 13.7 per 1000 patient-years, crude HR 1.58 (95% CI 1.46–1.71). But after adjustment for age, sex, ethnicity, socioeconomic status, smoking, alcohol abuse, obesity, diabetes mellitus, previous history of cancer and aspirin and statin use, there was no difference between the two groups (HR 0.93, 95% CI 0.86–1.01). The HR for the risk of cancer associated with AS was inversely proportional to age (*P* < 0.001 for the interaction between AS and age) (Fig. 3). Stratification analysis by age quartiles, revealed a significantly higher risk of cancer in the lowest age quartile (age 20–49.7 years), adjusted HR 1.91, 95% CI 1.08–3.39 (Table 2). Similarly, an increased cumulative incidence of cancer in AS was observed only in the lowest age quartile (Fig. 4). In this group of patients, the proportional hazard assumption was not violated.

**Table 2:** Crude and adjusted incidence rate of non-hematological cancers according to AS status and age

AS status	Events *(n)*	Follow-up *(person-years)*	Incidence rate *(Per 1000 p-y)*	Crude HR (95% CI)	Adjusted HR^**a**^ (95% CI)
***Entire study population (n = 105,897)***					
**Mod or Severe AS** *(n = 7013)*	677	29,800	22.7	**1.58 (1.46–1.71)**	0.93 (0.86–1.01)
**No AS** *(n = 98,884)*	10,028	733,067	13.7	Reference	Reference
***Age lowest quartile: 20–49.7 years (n = 26,476)***					
**Mod or Severe AS** *(n = 183)*	12	1330	9.0	**2.26 (1.28–3.99)**	**1.91 (1.08–3.39)**
**No AS** *(n = 26,293)*	887	223,466	4.0	Reference	Reference
***Age second quartile: 49.7–63 years (n = 26,477)***					
**Mod or Severe AS** *(n = 558)*	58	3820	15.2	1.24 (0.95–1.60)	1.15 (0.88–1.49)
**No AS** *(n = 25,919)*	2689	218,792	12.3	Reference	Reference
***Age third quartile: 63–74.7 years (n = 26,475)***					
**Mod or Severe AS** *(n = 1610)*	176	8345	21.1	1.02 (0.87–1.18)	0.96 (0.82–1.12)
**No AS** *(n = 24,865)*	3575	175,370	20.4	Reference	Reference
***Age highest quartile: > 74.7 years (n = 26,469)***					
**Mod or Severe AS** *(n = 4662)*	431	16,306	26.4	1.00 (0.91–1.11)	0.99 (0.89–1.10)
**No AS** *(n = 21,807)*	2877	115,438	24.9	Reference	Reference

^a^Adjusted for age (continuous variable), sex, ethnicity, socioeconomic status, smoking, alcohol abuse, obesity, diabetes mellitus, previous history of cancer and aspirin and statin use

*CI* confidence interval, *HR* hazard ratio, *p-y* patient-year

Since bicuspid aortic valve (BAV) is more frequent in younger patients with AS, we examined the association between BAV and cancer in patients without any degree of AS (*n* = 98,884). Overall, BAV was found in 629 (0.6%) subjects with no-AS compared to 534 (7.6%) with AS. The presence of BAV without AS was not associated with an increased risk of cancer (HR 1.05, 95% CI, 0.79–1.40).

We performed a sensitivity analysis to account for possible biases. First, we performed further adjustment for heart failure, ischemic heart disease, peripheral vascular disease, previous stroke, atrial fibrillation and year of study entry. This analysis showed similar results with significantly higher risk of cancer in the lowest age quartile (age 20–49.7 years), adjusted HR 1.86, 95% CI 1.04–3.30 (Table 3). Second, we restricted the analysis to patients without a history of cancer, since these patients may have an increased risk for other malignancies (Table 3). Patients with AS at the lowest age quartile had a significantly increased risk for cancer even after exclusion of prior cancer. Third, we did landmark analysis to assess a possible surveillance bias shortly after AS diagnosis, by excluding patients with a cancer diagnosis during the first year after the index echo (Table 3). In this analysis the magnitude of the effect of AS remained unchanged, but did not reach statistical significance.

**Table 3:** Sensitivity analysis displaying multivariable^a^ hazard ratios (HRs), stratified by age quartiles

Age quartiles	Adjustment for additional variables^b^ *(n = 105,897)* HR (95% CI)	Patients without prior malignancy^c^ *(n = 87,172)* HR (95% CI)	One year landmark analysis^d^ *(n = 95,930)* HR (95% CI)
**Lowest quartile** (20–49.7 years)	1.86 (1.04–3.30)	2.24 (1.06–4.71)	1.71 (0.88–3.31)
**Second quartile** (49.7–63 years)	1.14 (0.88–1.48)	0.91 (0.63–1.32)	1.22 (0.93–1.62)
**Third quartile** (63–74.7 years)	0.95 (0.82–1.11)	0.96 (0.79–1.16)	1.03 (0.88–1.22)
**Highest quartile** (> 74.7 years)	1.02 (0.92–1.13)	0.96 (0.85–1.09)	1.02 (0.90–1.16)

^a^Adjusted for age (continuous variable), sex, ethnicity, socioeconomic status, smoking, alcohol abuse, obesity, diabetes mellitus, previous history of cancer (except for patients without prior malignancy analysis) and aspirin and statin use

^b^In addition to the above variables, additional adjustment was performed for heart failure, ischemic heart disease, peripheral vascular disease, previous stroke, atrial fibrillation, and year of study entry

^c^Without prior malignancy of any type (hematological and non-hematological)

^d^ The landmark analysis excluded patients with cancer diagnosed in the first year and was restricted to patients with follow up greater than one year

Distribution of the different types of cancer in patients with and without AS is presented in Table 4.

**Table 4:** Distribution of invasive non-hematological malignancies

Invasive non-hematological malignancies type	No AS	AS
***Lowest age quartile: 20–49.7 years***		
Breast	230 (25.9%)	3 (25.0%)
Prostate	17 (1.9%)	0 (0%)
Gastrointestinal tract	83 (9.4%)	2 (16.7%)
Lung	62 (7.0%)	0 (0%)
Pancreas, liver and biliary tract	41 (4.6%)	0 (0%)
Kidney and bladder	59 (6.7%)	1 (8.3%)
Ovary, uterus and cervix	89 (10.0%)	1 (8.3%)
Melanoma	47 (5.3%)	1 (8.3%)
Thyroid	65 (7.3%)	2 (16.7%)
Other and unspecified site	194 (21.9%)	2 (16.7%)
***All other patients, age > 49.7 years***		
Breast	1144 (12.5%)	56 (8.4%)
Prostate	951 (10.4%)	54 (8.1%)
Gastrointestinal tract	1505 (16.5%)	137 (20.6%)
Lung	1105 (12.1%)	71 (10.7%)
Pancreas, liver and biliary tract	702 (7.7%)	62 (9.3%)
Kidney and bladder	1153 (12.6%)	99 (14.9%)
Ovary, uterus and cervix	442 (4.8%)	24 (3.6%)
Melanoma	507 (5.5%)	44 (6.6%)
Thyroid	179 (2.0%)	4 (0.6%)
Other and unspecified site	1453 (15.9%)	114 (17.1%)

## Discussion

In the present study, young patients in the lowest age quartile (20–49.7 years), who had moderate or severe AS, had a significantly higher adjusted risk of cancer compared to patients without AS. In addition, we have shown that age interacts with AS and modifies the risk for non-hematological malignancies. The risk of cancer associated with AS was inversely proportional to age. Our findings reconfirm our previous report in which the data was derived from a different study population [9]. In our earlier study, a post hoc analysis showed a significantly increased adjusted risk of non-hematological malignancies in younger patients with AS (40–60 years old), but not in older patients.

A cause-and-effect relationship between early cardiac remodeling secondary to left ventricular pressure overload and cancer was established using the TAC mice model [9]. Compared to controls, TAC-operated mice developed larger tumors after subcutaneous and fat-pad injection of lung and breast cancer cells. In addition, compared to sham operated animals, TAC operated mice showed enhanced tumor metastasis after intravenous injection of tumor cells. Periostin was identified as a putative mediator of this effect [9]. Periostin is a non-structural extracellular matrix protein secreted from the remodeled heart and associated with cancer progression and metastasis [17, 18]. Periostin mRNA levels in the heart increased after TAC, resulting high serum periostin levels. In vitro, serum from TAC-operated mice induced cancer cells proliferation, but depletion of periostin from the serum abrogated this effect. The effect of periostin on cancer progression needs to be confirmed in vivo.

It is not clear why the risk of cancer attributable to AS is most notable early in life and decreases with age. The incidence of both AS and cancer increases with age [19, 20]. In a population study from Tromsø, Norway, the prevalence of AS (defined as aortic mean gradient ≥15 mmHg) was 0.2% in the 50–59 year cohort, and increased exponentially with age to 9.8% in the 80–89 year cohort [21]. This phenomenon is due to the ongoing process of lipid deposition, inflammation and calcification of the aortic valve [22]. The incidence of invasive cancer peaks at age 70 but declines with advanced age (US population 2009 data) [19]. Cellular senescence can suppress cancer early in life, but later in life it may promote hyperplastic pathology and cancer by the secretion of chemokines and growth factors from senescent cells, possibly through the promotion of chronic inflammation [23]. The effect of tumor suppression by cellular senescence could be more dominant at advanced age, causing the decline in the incidence of cancer late in life. It is also possible, that while cell senescence and other disease processes promoting cancer are dominant in older age, periostin secretion as a result of cardiac adaptive remodeling secondary to AS is more dominant in younger age.

We performed a sensitivity analysis, by excluding patients with a history of cancer that could be at a higher risk for recurrent cancer. Young patients with AS had a significantly higher adjusted risk for cancer even after excluding patients with prior cancer. To exclude a surveillance bias, we excluded from the analysis patients in whom cancer was diagnosed during the first year after the diagnosis of AS. Albeit not reaching statistical significance, the magnitude of the effect of AS on cancer risk remained unchanged in younger age, arguing against a surveillance bias.

While most patients with AS above age 70 have a tricuspid aortic valve, almost all patients with AS younger than 50 have congenital AS, usually BAV [24]. It is possible that the increased risk of cancer is associated with BAV, but to the best of our knowledge there are no reports of such association. Furthermore, in our study, no association was found between BAV and cancer in patients without AS. Furthermore, the TAC mice model argues in favor of pressure overload secondary to AS and adaptive cardiac remodeling as the cause of cancer.

Our findings may suggest a closer cancer surveillance approach in young patients with AS, however the impact of such an approach on patient outcome is unknown. Early surgery in young patients with severe AS may decrease the risk for cancer, but should be weighed against the inherent risks of a mechanical prosthetic valve in this age group, mainly bleeding and thromboembolic complications [25, 26].

## Limitations

In this study we rely on data retrieved from a computerized database originally designed for purposes of administrative and clinical management and not specifically designed for the present study. Yet, the database is periodically updated with data from the Israeli National Cancer Registry (INCR), which is highly accurate [13]. Based on the existence of strict criteria and standardized echocardiographic methodology for AS assessment, we estimate that the data on AS is highly accurate. Hence, it is likely that misclassification of the two main study variables (AS and cancer) is small.

In addition, our study was limited to patients referred for echocardiography in a single tertiary medical center, and therefore includes a selected population, limiting the generalizability of our findings. In order to determine the risk for cancer in young patients with AS in a prospective population study, a very large sample size will be needed. The sample size of our study, together with the adjustment for a large number of confounders, and the concordance with our previous publication which was based on a different large database from another medical center, suggests that our findings are valid. Yet, due to the observational nature of our study residual confounders remain a major concern. We could not control for other potential confounders such as chronic inflammation, radiation, immunosuppression and diet which are known risk factors for cancer and might differ between subjects with and without AS.

In the current study we have focused on the association between AS and non-hematological malignancies. Our findings may not apply to hematological malignancies.

## Conclusions

Our study suggests that younger patients with moderate or severe AS may have an increased risk of non-hematological malignancies. The risk of cancer associated with AS appears to be inversely proportional to age. These data are consistent with our previous findings from a different database, and together with the murine TAC model results may suggest that AS increases the risk of cancer.

## Data Availability

The datasets used and/or analyzed during the current study are available from the corresponding author on reasonable request.
